# Improving the clinical outcomes by extended culture of day 3 embryos with low blastomere number to blastocyst stage following frozen–thawed embryo transfer

**DOI:** 10.1007/s00404-020-05774-1

**Published:** 2020-10-08

**Authors:** Bo Li, Jianlei Huang, Li Li, Xiao He, Ming Wang, Hengde Zhang, Yuping He, Bin Kang, Yongqian Shi, Shuqiang Chen, Xiaohong Wang

**Affiliations:** Department of Obstetrics and Gynecology, Tangdu Hospital, The Air Force Military Medical University, Xi’an, 710038 China

**Keywords:** Retarded embryos, Blastocyst culture, Implantation rate, Clinical pregnancy rate, Abortion rate

## Abstract

**Purpose:**

This study aimed to investigate whether the extended culture of day 3 (D3) embryos with low blastomere number to blastocyst following frozen–thawed embryo transfer improved the clinical outcomes.

**Methods:**

This was a retrospective study of clinical data of women undergoing in vitro fertilization/intracytoplasmic sperm injection (IVF/ICSI) cycles in the Tangdu Hospital. The patients were divided into groups with 4–5, 6, 7–9 and > 9 cells based on the blastomere number of D3 embryos. The clinical outcomes were compared.

**Results:**

In fresh transfer cycles, the implantation and clinical pregnancy rates significantly decreased, while the abortion rate significantly increased in the groups with 4–5 and 6 cells compared with those with 7–9 and > 9 cells. In frozen–thawed transfer cycles, the clinical pregnancy and implantation rates for a single blastocyst transfer cycle showed no significant differences in the groups with 4–5 and 6 cells compared with those with 7–9 and > 9 cells. However, the abortion rate was significantly higher in the group with 4–5 cells than in that with 7–9 and > 9 cells. In the double blastocyst transfer cycle, the clinical pregnancy rate showed no significant differences among the groups with 4–5, 6, and 7–9 cells.

**Conclusion:**

The implantation and clinical pregnancy rates of D3 embryos with 6 cells significantly decreased; these embryos were not considered as high-quality embryos. Extended culture of D3 embryos with ≤ 6 blastomeres to blastocysts, particularly 6-cell embryos, resulted in a similar clinical pregnancy rate as that of blastocysts derived from D3 embryos with ≥ 7 blastomeres.

## Introduction

Embryos obtained from assisted reproductive technology (ART) are transferred into a woman’s uterus on day 3 after egg collection (D3). For achieving satisfactory pregnancy outcomes, embryos are usually graded according to the standardized scoring criteria for transfer. Currently, the criteria for evaluating embryo quality mainly depend on the number of blastomeres, degree of fragmentation, evenness of blastomeres, and other morphological indicators [1]. The number of blastomeres on D3 represents the cleavage rate of embryos and is regarded as the most critical indicator for predicting the developmental potential of embryos and pregnancy outcomes [1–3].

Studies have shown that D3 embryos with low blastomere number have significantly reduced developmental potential, negatively influencing the pregnancy rates [1–3]. Zhang et al. reported that D3 embryos with six, seven, and eight cells were good-quality embryos. They also demonstrated that D3 embryos with less than six blastomeres were associated with low clinical pregnancy rate and increased abortion rate [4]. Alikani et al. found that the embryos with seven to nine blastomeres had higher developmental potential and blastocyst formation rate compared with embryos having less than seven blastomeres or more than nine blastomeres [5]. Kong et al. also indicated that ≥ 7 blastomeres in the embryos showed higher developmental potential, which decreased for ≤ 6 blastomeres in the embryos [2]. These results indicated that the transfer of D3 embryos with lower mean number of blastomeres significantly resulted in reduced implantation and clinical pregnancy rates and increased abortion rate [2, 6]. However, different classifications were forwarded on whether D3 six-cell embryos were good- or poor-quality embryos. Some studies regarded D3 six-cell embryos as good-quality embryos [4, 7], but few others showed them to be associated with low clinical pregnancy rates and increased abortion rates [2, 7].

Recent advancements in embryo culture media have led to a shift in ART practice from cleavage-stage embryo transfer to blastocyst-stage transfer [8]. Moreover, the transfer of blastocyst-stage embryos could improve the implantation rate, live birth rate, and other associated outcomes compared with embryo transfers in the cleavage stage [9]. However, information regarding the blastocyst rate and subsequent clinical outcomes in frozen embryo transfer cycles of D3 embryos with cell number ≤ 6 is scarce. This study mainly aimed to retrospectively analyze the clinical outcomes of the transfer of D3 embryos with cells ≤ 6 or blastocysts derived from extended culture of these embryos to determine whether D3 six-cell embryos had high developmental potential and whether the transfer of blastocysts by extended culture could substantially increase the chances for a successful pregnancy.

## Materials and methods

### Participants

The clinical data of patients undergoing in vitro fertilization/intracytoplasmic sperm injection (IVF/ICSI) cycle for assisted pregnancy in the reproductive medicine center of Tangdu Hospital from January 2013 to December 2016 were retrospectively analyzed. Patients underwent controlled ovarian stimulation during natural or microstimulation cycles.

### Methods

Controlled ovarian stimulation was performed according to the standard long and short antagonist protocols at the center. The dominant follicles achieved a diameter of ≥ 18 mm or at least three follicles had a diameter of ≥ 16 mm after administering 5000–10,000 U of human chorionic gonadotropin (HCG, Zhuhai, lizhu) or 250 μg Aize (Syrano, Switzerland) by intramuscular injection according to the estrogen levels. After 34–36 h, oocyte retrieval was performed with a transvaginal aspiration needle under the guidance of vaginal ultrasound.

Embryo culture and evaluation: IVF/ICSI technique was used for in vitro fertilization, and the fertilization results were evaluated 16–18 h after fertilization. Normal fertilized zygotes were cultured using a desktop three-gas incubator (COOK, K-MINC-1000) at 37 °C in the presence of 6% CO_2_, 5% O_2_, and 89% N_2_. According to the Istanbul embryo consensus, D3 embryo observation and evaluation were carried out after fertilization (68 ± 1 h) and blastocyst development was evaluated after fertilization (116 ± 2 h) [10]. This was followed by the selection of high-quality embryos for transfer. If no high-quality embryos were obtained, the embryos with more blastomeres, less fragments, and no obvious unevenness of blastomeres were selected.

Embryo evaluation criteria: D3 embryos in the cleavage stage were graded according to the standard of morphological grading in the laboratory: grade I: blastomere number in the embryos was > 6, blastomeres were even, and fragmentation was ≤ 5%; grade II: blastomere number in the embryos was > 6, blastomeres were slightly uneven, and fragmentation was ≤ 20%; grade III: blastomere number in the embryos ranged from 4 to 6, blastomeres were uneven, and fragmentation was between 20 and 50%; and grade IV: blastomere number in the embryos was < 4 and fragmentation was > 50%. The two-step scoring criteria proposed by Garden were adopted for grading blastocysts [11]: high-quality blastocysts were at stage 4 or higher of blastocyst expansion, with a scoring of 4BB or higher; and available blastocysts were at stage 3 or higher of blastocyst expansion, with a scoring of 3CC or higher.

Corpus luteum support and monitoring: After embryo transplantation, human chorionic gonadotropin (HCG) or progesterone was used to maintain the corpus luteum function, and blood HCG was detected on day 15. If HCG was positive, ultrasound examination was performed 4 weeks after the transfer and those with fetal heart beat were diagnosed with clinical pregnancy.

In this study, the D3 embryos were divided into groups with 4–5, 6, 7–9, and > 9 cells according to the number of blastomeres. First, the clinical pregnancy rate, implantation rate, and abortion rate were analyzed among groups in the fresh cycle. Second, the blastocyst formation rate, high-quality blastocyst rate, and available blastocyst rate were compared. Finally, the embryo implantation rate, clinical pregnancy rate, abortion rate, and other indicators of frozen–thawed cycle transfer of D3 embryos with different development rates after extending the culture to blastocysts were analyzed and compared.

### Statistical analysis

SPSS16.0 statistical software was used for statistical analysis. The measurement data were expressed as mean ± standard deviation ($\overline{x}$ ± *s*). Analysis of variance (ANOVA) was performed to compare between groups. A *P* value < 0.05 was considered to be statistically significant. The counting data were expressed as percentage (%). The comparison between groups was conducted using the chi-square test, and the difference was statistically significant if the *P* value was < 0.05.

## Results

### Comparison of general information

This study included 6331 IVF/ICSI cycles for assisted pregnancy. The clinical characteristics were compared. The results showed that age, infertility duration, Gn dosage, and days of ovulation induction of the patients were not significantly different (*P* ≥ 0.05) among the four groups. Moreover, no significant difference was found in the number of retrieved oocytes per cycle between the group with 4–5 cells (9.79 ± 5.31) and the other groups (*P* ≥ 0.05), while the number of retrieved oocytes was significantly higher in the group with 7–9 cells (10.71 ± 4.46) and > 9 cells (9.89 ± 5.22) than in the groups with 6 cells (8.11 ± 5.23) (*P* < 0.05; Table 1).

**Table 1:** Comparison of general data of patients ($\overline{x}$ ± *s*)

Group	Cycle number	Maternal age	Paternal age	Infertility duration (years)	Gn dosage	Days of ovulation induction	Average number of oocytes
4–5 cells	33	30.73 ± 4.07	32.15 ± 4.58	4.11 ± 2.91	33.40 ± 15.86	10.82 ± 2.19	9.79 ± 5.31^abc^
6 cells	79	31.74 ± 5.68	33.19 ± 5.99	4.68 ± 4.03	32.48 ± 17.94	10.59 ± 3.07	8.11 ± 5.23^a^
7–9 cells	5988	31.12 ± 4.83	32.91 ± 5.59	4.29 ± 3.33	30.03 ± 15.51	10.99 ± 2.54	10.71 ± 4.46^b^
> 9 cells	231	31.53 ± 5.05	33.32 ± 5.64	4.59 ± 4.55	30.00 ± 16.17	10.57 ± 2.96	9.89 ± 5.22^c^

Different shoulder letters in the same column mean significant difference (*P* < 0.05), while the same letter means insignificant difference (*P* ≥ 0.05)

### Pregnancy outcomes after fresh embryo transfer according to the number of blastomeres in the embryos on D3

The clinical pregnancy rates were 36.36, 27.85, 54.71, and 54.11 (%); the implantation rates were 21.21, 17.24, 38.06, and 37.50 (%); and the abortion rates were 8.33, 31.82, 13.86, and 12.00 (%), respectively, in the groups with 4–5, 6, 7–9, and > 9 cells after fresh embryo transfer on D3 (Table 2). The analysis of pregnancy outcomes of embryos in fresh transfer cycle in different groups showed no significant differences in the clinical pregnancy rate, implantation rate, and abortion rate between the groups with 4–5 and 6 cells (*P* ≥ 0.05). Meanwhile, no significant differences were observed in the clinical pregnancy rate, implantation rate, and abortion rate between the groups with 7–9 and > 9 cells (*P* ≥ 0.05). The clinical pregnancy rates were significantly lower in the groups with 4–5 and 6 cells than in the group with 7–9 cells (*P* < 0.05), while no significant difference was observed between the groups with 4–5 and > 9 cells (*P* ≥ 0.05). The clinical pregnancy rate significantly decreased in the group with 6 cells compared with the group with > 9 cells (*P* < 0.05). The implantation rate was significantly lower in the groups with 4–5 and 6 cells than in those with 7–9 and > 9 cells (*P* < 0.05). The abortion rate sharply increased in the group with 6 cells than in the groups with 4–5, 7–9, and > 9 cells (*P* < 0.05), while no significant difference in abortion rate was observed between the groups with 4–5, 7–9, and > 9 cells (*P* ≥ 0.05).

**Table 2:** Analysis of pregnancy outcomes after fresh transfer with the embryos with different blastomeres number in D3

Group	Cycles	Clinical pregnancy rate (%)	OR (95% CI)	Implantation rate (%)	OR (95% CI)	Abortion rate (%)	OR (95% CI)
4–5 cells	33	36.36^ab^	1	21.21^a^	1	8.33^ab^	1
6 cells	79	27.85^b^	0.68 (0.29–1.60)	17.24^a^	0.77 (0.37–1.61)	31.82^b^	5.13 (0.55–47.9)
7–9 cells	5988	54.71^c^	2.11 (1.04–4.30)	38.06^b^	2.28 (1.26–4.12)	13.86^a^	1.77 (0.23–13.7)
> 9 cells	231	54.11^ac^	2.06 (0.97–4.39)	37.50^b^	2.23 (1.20–4.15)	12.00^a^	1.50 (0.18–12.5)

Different shoulder letters in the same column mean significant difference (*P* < 0.05), while the same letter means insignificant difference (*P* ≥ 0.05)

### Developmental potential of embryos with different number of blastomeres

The blastocyst formation rate, high-quality blastocyst rate, and available blastocyst rate were compared among the groups with 4–5, 6, 7–9, and > 9 cells to explore the development potential of embryos with different blastomere numbers. The results revealed that the blastocyst formation rate increased with cell number by 28.17%, 41.73%, 53.83%, and 58.27% in embryos with 4–5, 6, 7–9, and > 9 cells, respectively (*P* < 0.01; Table 3). The good-quality blastocyst formation rate also increased with cell number by 9.77%, 12.59%, 29.85%, and 37.89% in embryos with 4–5, 6, 7–9, and > 9 cells, respectively (*P* < 0.01; Table 3). Meanwhile, the available blastocyst rate increased with cell number by 7.11%, 12.98%, 30.69%, and 36.35% (*P* < 0.01; Table 3) in the groups with 4–5, 6, 7–9, and > 9 cells, respectively. These results indicated that the developmental potential significantly increased with cell number on D3. In addition, the blastocyst formation rate of 6-cell embryos reached 41.73%, indicating that the 6-cell embryos have high developmental potential (Table 3).

**Table 3:** The developmental potential of D3 embryo with different blastomeres number by extended culture

Group	Embryo number	Blastocyst formation rate (%)	OR (95% CI)	High quality blastocyst rate (%)	OR (95% CI)	Available blastocyst rate (%)	OR (95%CI)
4–5 cells	12,426	28.17^a^	1	9.77^a^	1	7.11^a^	1
6 cells	8148	41.73^b^	1.83 (1.72–1.94)	12.59^b^	1.33 (1.14–1.55)	12.98^b^	1.95 (1.78–2.14)
7–9 cells	15,290	53.83^c^	2.97 (2.83–3.13)	29.85^c^	3.93 (3.48–4.44)	30.69^c^	5.79 (5.36–6.25)
> 9 cells	3012	58.27^d^	3.56 (3.28–3.87)	37.89^d^	5.63 (4.86–6.53)	36.35^d^	7.47 (6.75–8.26)

Different shoulder letters in the same column mean significant difference (*P* < 0.05), while the same letter means insignificant difference (*P* ≥ 0.05)

### Pregnancy outcomes of single blastocyst transfer after extended culture of embryos with different numbers of blastomeres

Next, the pregnancy outcomes of frozen–thawed cycle of embryos with different blastomere numbers after extended culture to blastocysts following single blastocyst transfer were analyzed. The clinical pregnancy rate and implantation rate were 61.36%, 62.79%, 66.80%, and 72.79% in the groups with 4–5, 6, 7–9, and > 9 cells, respectively (*P* ≥ 0.05; Table 4), with no significant difference. The abortion rate of blastocysts was significantly higher in the group with 4–5 cells than in that with 7–9 cells (*P* < 0.05; Table 4). However, the abortion rate of blastocysts in the group with 6 cells showed no significant differences compared with groups with 7–9 and > 9 cells (*P* ≥ 0.05; Table 4).

**Table 4:** Pregnancy outcomes of single blastocyst transfer after extended culture of D3 embryos with different blastomeres number

Group	Cycles	Clinical pregnancy rate (%)	OR (95% CI)	Implantation rate (%)	OR (95% CI)	Abortion rate (%)	OR (95% CI)
4–5 cells	44	61.36	1	61.36	1	29.63^a^	1
6 cells	43	62.79	1.06 (0.45–2.53)	62.79	1.06 (0.45–2.53)	22.22^ab^	0.68 (0.20–2.32)
7–9 cells	998	66.80	1.27 (0.68–2.36)	66.80	1.27 (0.68–2.36)	13.94^b^	0.39 (0.16–0.90)
> 9 cells	136	72.79	1.69 (0.82–3.44)	72.79	1.69 (0.82–3.44)	18.18^ab^	0.53 (0.20–1.39)

Different shoulder letters in the same column mean significant difference (*P* < 0.05), while the same letter means insignificant difference (*P* ≥ 0.05)

### Pregnancy outcomes of double blastocyst transfer after extended culture of embryos with different blastomere number

The pregnancy outcomes of frozen–thawed cycle of embryos with different blastomere number after extended culture to blastocysts following double blastocyst transfer were also compared. The clinical pregnancy rate was 64.44%, 73.17%, 71.98%, and 81.61% in the groups with 4–5, 6, 7–9, and > 9 cells (Table 4). The pregnancy outcome of double blastocyst transfer in frozen–thawed cycles showed no significant differences in clinical pregnancy rate among the groups with 4–5, 6, and 7–9 cells (*P* ≥ 0.05; Table 4), whereas the clinical pregnancy rate significantly increased in the group with > 9 cells than in that with 4–5 cells (*P* < 0.05; Table 4). Furthermore, the implantation rate significantly increased in the groups with 7–9 and > 9 cells than in the group with 4–5 cells (39.78%, 52.91%, and 66.67%, respectively) (*P* < 0.05; Table 4). However, no significant differences were observed between the groups with 4–5 and 6 cells (39.78% vs 48.54; *P* ≥ 0.05). The implantation rate significantly increased in the group with > 9 cells than in that with 6 cells (*P* < 0.05; Table 4). No significant differences were found in the abortion rate among the groups with 4–5, 6, and 7–9 cells compared with the group with 4–5 cells. The abortion rate significantly decreased in the group with > 9 cells than in that with 4–5 cells (*P* < 0.05), but no significant differences were noted in the abortion rate between the groups with 6, 7–9, and > 9 cells (*P* ≥ 0.05; Table 5).

**Table 5:** Pregnancy outcomes of double blastocysts transfer after extended culture of D3 embryos with different blastomeres number

Group	Cycles	Clinical pregnancy rate (%)	OR (95% CI)	Implantation rate (%)	OR (95% CI)	Abortion rate (%)	OR (95% CI)
4–5 cells	45	64.44^a^	1	39.78^a^	1	20.69^a^	1
6 cells	82	73.17^ab^	1.51 (0.69–3.29)	48.54^ab^	1.43 (0.86–2.38)	23.33^a^	1.17 (0.40–3.43)
7–9 cells	514	71.98^ab^	1.42 (0.75–2.69)	52.91^b^	1.70 (1.10–2.62)	14.59^a^	0.66 (0.26–1.68)
> 9 cells	87	81.61^b^	2.45 (1.08–5.54)	66.67^c^	3.03 (1.80–5.10)	7.04^b^	0.29 (0.08–1.04)

Different shoulder letters in the same column mean significant difference (*P* < 0.05), while the same letter means insignificant difference (*P* ≥ 0.05)

## Discussion

The present study demonstrated that the fresh transfer of D3 embryos with 4–5 and 6 cells resulted in low clinical pregnancy and implantation rates and high abortion rate compared with that with D3 embryos with 7–9 and > 9 cells. In addition, no significant differences were observed in the clinical pregnancy rate and implantation rate between embryos with 4–5 and 6 cells. Also, the blastocyst formation rate in D3 6-cell embryos was 41.73%, which was lower than that obtained in embryos with 7–9 and > 9 cells, but higher than that in embryos with 4–5 cells. The transfer of single blastocysts from the extended culture of D3 embryos with 4–5 and 6 cells resulted in similar clinical pregnancy rate and implantation rate compared with embryos with 7–9 and > 9 cells. The transfer of double blastocysts from the extended culture of D3 developmentally retarded embryos also resulted in equivalent clinical pregnancy rates compared with embryos with 7–9 cells. These results suggested that the extended culture of D3 embryos with low blastomere number, particularly the 6-cell embryos, to blastocysts showed similar clinical pregnancy rates compared with blastocysts derived from D3 embryos with 7–9 and > 9 cells.

A low blastomere number of D3 embryos commonly indicated poor clinical outcomes [2, 6, 12]. The transfer of D3 four-cell embryos resulted in clinical pregnancies and live births, albeit a low rate [6]. According to time-lapse research, the implantation rate and live birth rate significantly decreased in D3 embryos with < 5 and 5–6 blastomeres compared with those with 7–10 blastomeres; the implantation rate and live birth rate of embryos with > 10 blastomeres were the highest [2]. However, Zhang et al. demonstrated that D3 embryos with < 6 blastomeres had decreased clinical pregnancy rates and increased abortion rates [4]. In addition, some studies indicated that D3 6-cell embryos were good-quality embryos with high developmental potential [4, 7]. In the present study, the transfer of D3 embryos with 4–5 and 6 cells resulted in a significant decrease in both implantation and clinical pregnancy rates and an increase in abortion rates compared with those with 7–9 and > 9 cells. However, these results demonstrated no significant differences in the clinical pregnancy rate, implantation rate, and abortion rate between embryos with 6 and 4–5 cells. The D3 embryos with 6 cells did not have high developmental potential compared with those with 4–5 cells, and might not be classified as good-quality embryos. The main reasons for poor clinical outcomes of D3 embryos with ≤ 6 blastomeres might be partly due to the embryo-endometrium asynchrony. The endometrium became receptive as a result of series of temporal and spatial hormonal events, contributing to the success of embryo implantation [13–15]. D3 embryos with 7–9 blastomeres demonstrated high implantation and pregnancy rates because their development was consistent with implantation window period. However, the embryonic development of D3 embryos with ≤ 6 blastomeres was delayed than the endometrium receptivity and missed the implantation window, resulting in a decline in implantation. This embryo-endometrium asynchrony could be overcome by thawing the frozen developmentally delayed cleavage-stage embryos on D3 for 24 h in advance and choosing to restore the mitotic embryos for transfer, thus increasing the implantation and pregnancy rates [16, 17].

Also, the blastocyst formation rate, high-quality blastocyst rate, and available blastocyst rate increased with blastomere number of D3 embryos. These results also indicated that the developmental potential of D3 embryos increased with the number of blastomeres. The blastocyst formation rate, high-quality blastocyst rate, and available blastocyst rate of D3 embryos with ≤ 6 blastomeres significantly decreased compared with embryos with more blastomeres. However, the blastocyst formation rate of D3 embryos with 6 cells was still considerable. Zhu et al. demonstrated that the blastocyst formation rate of D3 embryos with 6 cells reached 47.87% after extended culture, which was higher than that of embryos with 4–5 cells [7]. Kong et al. reported that the blastocyst formation rate of embryos with 5–6 cells was more than 40%, while the blastocyst formation rate of embryos with < 5 blastomeres was only 20.7% [2]. Similarly, Shapiro et al. showed that the blastocyst formation rate of D3 embryos with 6 and 5 blastomeres was 41.7% and 39%, respectively [18]. The results also confirmed that the blastocyst formation rate of D3 embryos with 6 cells was up to 41.73%, but it was only 28.17% for those with 4–5 cells. The blastocyst formation rate, high-quality blastocyst rate, and available blastocyst rate sharply decreased in embryos with 4–5 cells compared with those with 6 cells. The decreased developmental potential of D3 embryos with ≤ 6 blastomeres might be due to an increase in the chromosomal anomalies [19–21]. It was evident that D3 embryos with low blastomere numbers were at significantly elevated risk of aneuploidy [22]. Other studies based on trophectoderm biopsy and chromosomal analysis showed high aneuploidy rates in slow-developing blastocysts [23]. These studies demonstrated an association between chromosomal abnormality and embryo developmental rate. The results confirmed that D3 embryos with 6 cells showed a considerable blastocyst formation rate, indicating that the transfer of vitrified and warmed blastocysts developed from these D3 embryos might increase the chances of successful pregnancy.

Previous studies showed that the extended culture of D3 poor-quality embryos into blastocysts was then transferred into frozen–thawed cycle, resulting in good clinical pregnancy and implantation rates [24, 25]. After extended culture of embryos with poor developmental potential into blastocysts, the clinical pregnancy rate could reach 24.6% and the cumulative live birth rate increased from 43 to 47% [26]. The results showed no significant differences in the clinical pregnancy rate and implantation rate among the groups with 4–5, 6, 7–9, and > 9 cells in the single-blastocyst transfer cycle. However, the abortion rate of blastocysts derived from embryos with 4–5 cells was significantly higher than that derived from embryos with 7–9 cells. In the double-blastocyst transfer cycle, no significant differences were observed in the clinical pregnancy rate among the groups with 4–5, 6, and 7–9 cells. Although the implantation rate significantly decreased in blastocysts derived from the groups with 4–5 and 6 cells than in the group with 7–9 cells, equivalent clinical pregnancy outcomes occurred. These results demonstrated that blastocyst transfer derived from D3 embryos with ≤ 6 blastomeres resulted in similar clinical outcomes as embryos with > 6 blastomeres in either single- or double-blastocyst transfer cycles. Furthermore, the clinical pregnancy rate of single- and double-blastocyst transfer cycles of groups with 4–5 and 6 cells was > 60%, significantly higher than that of all four groups in fresh transfer cycles. These results indicated that extended culture provided an opportunity to screen high-quality embryos and eliminated the embryos with chromosomal abnormalities and/or poor developmental potential [21, 27]. Moreover, blastocyst transfer could avoid embryo-endometrium asynchrony, resulting in high clinical pregnancy rates compared with the fresh cycle [15, 28].

In conclusion, these results demonstrated that D3 embryos with ≤ 6 blastomeres were associated with low implantation and clinical pregnancy rates and high abortion rates compared with those with 7–9 and > 9 blastomeres. The extended culture of embryos with ≤ 6 blastomeres to blastocysts was then transferred to frozen–thawed cycle, resulting in good clinical pregnancy and implantation rates. These results indicated that the extended culture of D3 embryos with ≤ 6 blastomeres to blastocysts following frozen–thawed embryo transfer was an economical and effective method, improving the clinical pregnancy outcomes and relieving the economic burden and psychological pressure of patients [24, 26, 29]. The findings revealed that embryos with low blastomere number performed well in extended culture and frozen embryo transfer cycles.

However, this study still had some limitations. First, the data were retrospectively analyzed and the D3 fresh and extended culture warming cycles were not randomized. Second, the pre-implantation embryo stage is a vital period of epigenetic reprogramming, and the risk of abnormal imprinting may increase with the extension of in vitro operation and culture [30–32]. Whether extended culture interferes with embryonic imprinting and causes developmental defects still remains unclear. However, studies have shown that blastocyst stage transfer was associated with increased risks of preterm birth, very preterm birth, and large for gestational age [9, 33]. Finally, blastocyst-stage embryo transfer is associated with a risk of losing some embryos because these embryos may not survive in extended culture in vitro, but may survive in vivo if transferred on D3 [34]. Patients with fewer eggs have a high risk of no embryo transfer after extended culture. Therefore, it is not recommended to select extended culture for patients with few embryos, and embryo transfer should be carried out on D3 to improve the embryo utilization rate.
